# Comparing the clinical outcomes of an innovative bi-frame external fixation device compared to those of volar locking plate and external fixator device among patients with unstable distal radius fractures: a two-year retrospective comparative cohort study

**DOI:** 10.1080/07853890.2025.2524090

**Published:** 2025-06-26

**Authors:** Zhi-Yuan Yao, Xu‑Song Li, Jie-Feng Huang

**Affiliations:** a Department of Orthopaedics & Traumatology, The First Affiliated Hospital of Zhejiang Chinese Medical University (Zhejiang Provincial Hospital of Chinese Medicine), Hangzhou, China; b Department of Orthopaedics & Traumatology, Zhongshan Hospital of Traditional Chinese Medicine, Zhongshan, China

**Keywords:** Distal radius fracture, bi-frame external fixation device, external fixation, volar locking plate, clinical outcomes

## Abstract

**Background:**

Volar locking plate (VLP) fixation for unstable distal radius fractures (DRF) requires extensive soft tissue dissection and is associated with implant-related complications. Conventional external fixator (EF) carries risks such as reduction loss, pin loosening, and unstable traction. This study retrospectively evaluates the clinical efficacy and safety of a novel bi-frame external fixation device (BEF) compared with VLP and EF over a two-year period.

**Methods:**

rA total of 131 patients with unstable DRF treated between 2015 and 2022 at the First Affiliated Hospital of Zhejiang Chinese Medical University were included (42 BEF, 44 EF, 45 VLP). Functional outcomes included wrist range of motion and grip strength. Patient-reported outcomes were assessed using the Visual Analog Scale (VAS), Patient-Rated Wrist Evaluation (PRWE), and Quick Disabilities of the Arm, Shoulder, and Hand (QuickDASH) scores. Radiographic parameters and complications were recorded. Statistical comparisons used t-tests, Mann-Whitney U-tests, and chi-square or Fisher’s exact tests.

**Results:**

Baseline characteristics were comparable. BEF showed significantly shorter time from injury to surgery than VLP (17.2±5.2 vs. 68.3±24.1 hours, *p* < 0.001), and shorter operation time than EF (*p* = 0.028) and VLP (*p* < 0.001). Early outcomes favored VLP, but long-term results were comparable (*p* > 0.05). Compared with EF, BEF better preserved radial height, ulnar variance, pronation, and ulnar deviation (all *p* < 0.05), with fewer overall complications (*p* = 0.005).

**Conclusions:**

BEF represents an innovative, viable and safe alternative for unstable DRF. Further multicenter randomized trials with extended follow-up are warranted.

## Introduction

Distal radius fractures (DRF) are the most common fractures of the upper limb, accounting for over 16% of all fractures [1,2]. Unstable DRF may result from high-energy trauma in younger individuals or low-energy trauma in the elderly [3–5].

Multiple treatment options exist for unstable DRF, including casting, splinting, percutaneous Kirschner wire fixation, locking plates, bridge plates, external fixators, and arthroscopy. However, no single method has proven universally optimal, and the best treatment approach remains a subject of ongoing debate [6–9]. Among surgical techniques, volar locking plate (VLP) fixation and external fixation (EF) are the most commonly used [6,10].

VLP fixation generally provides favorable clinical and radiographic outcomes and facilitates early functional recovery [11,12]. Nonetheless, it is associated with complications such as tenosynovitis, median nerve injury, radial venous injury, and tendon injury. Reported overall complication rates for VLP reach 27%, including tenosynovitis (11.4%), tendon rupture (3.5%), carpal tunnel syndrome (2.6%), and complex regional pain syndrome (4.4%) [13,14]. Furthermore, VLP is linked to longer intervals from injury to surgery (65.9 ± 23.2 h), prolonged surgical times (79 ± 13 min), extended hospital stays (7 ± 2 days), and increased intraoperative blood loss (50 ± 24 mL), compared with EF [13,14]. Some cases also require additional surgery for implant removal [14].

EF has also demonstrated favorable outcomes in the management of unstable DRF [15]. When combined with Kirschner wires, EF can enhance stability and facilitate fracture reduction. Additionally, its components can often be removed in an outpatient setting [16]. Despite these advantages, EF has notable limitations, including a high incidence of reduction loss and fracture re-displacement due to the asymmetric forces applied by the uni-frame external fixator, particularly in osteoporotic bone [15]. Sanders et al. reported a malunion rate of 20% and a reduction loss rate of 50%, with pin loosening and pin breakage each occurring in 5.7% of cases [15].

**Figure 1.**
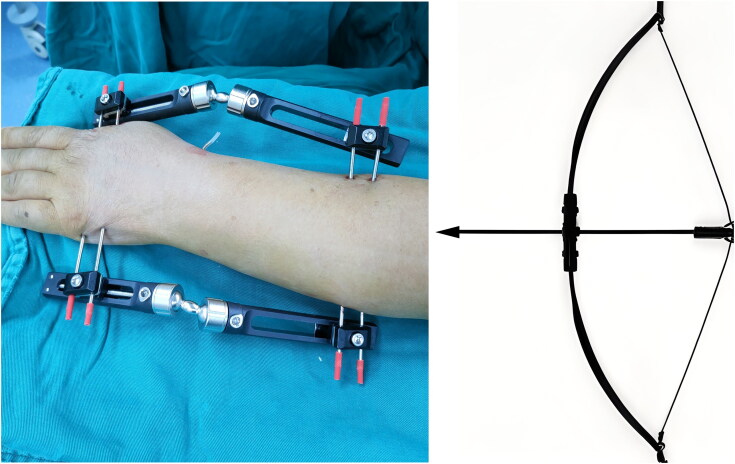
Illustration of the “bowstring effect” in BEF. The elastic deformation of Kirschner wires under applied tension generates a balanced traction force, facilitating controlled fracture reduction and stabilization. This mechanism optimizes mechanical stability while allowing micromotion conducive to secondary bone healing. BEF: bi-frame external fixation.

To overcome these limitations, we developed an innovative bi-frame external fixation device (BEF). The BEF shares structural similarities with the bidirectional traction devices proposed by Deng et al. and Chang et al. for the management of bicondylar tibial plateau fractures [17,18], and has demonstrated efficacy in reducing and maintaining complex tibial plateau fractures [19]. The BEF system consists of four Kirschner wires and two external fixators placed on the radial and ulnar sides, forming a closed-loop construct. When the knobs on the device are rotated, substantial longitudinal traction forces are generated, which are counteracted by the resistance provided by the Kirschner wires. This balance of traction and countertraction facilitates fracture reduction, restoring radial height and correcting fragment displacement *via* skeletal traction, ligamentotaxis, and soft-tissue compression [17,18]. The resultant "bowstring effect" induces slight elastic deformation of the Kirschner wires, ensuring stable and sustained traction for reduction and fixation (Figure 1). Additional 1.5 mm Kirschner wires are used to further enhance fixation stability and reduce the likelihood of postoperative displacement.

Compared with conventional EF, the BEF provides more effective, sustained, and symmetrical traction forces, which can improve fracture reduction and correction of fragment displacement while minimizing complications such as pin loosening, breakage, and loss of reduction. Additionally, BEF allows for closed fracture reduction without the need for open exposure, offering a less invasive alternative to VLP. This design may help mitigate complications associated with both techniques by reducing trauma-related issues observed with VLP, such as tenosynovitis, tendon injuries, and carpal tunnel syndrome, while improving fixation stability and reducing pin-related complications commonly encountered with EF.

To assess the clinical efficacy and safety of BEF in the treatment of unstable DRF, we conducted a two-year retrospective comparative cohort study comparing BEF with both VLP and EF.

## Methods

### Study design and setting

This retrospective comparative cohort study was conducted in the Department of Orthopedics at the First Affiliated Hospital of Zhejiang Chinese Medical University. It included all patients who underwent surgical treatment for unstable DRF between February 2015 and January 2022. Surgical intervention was indicated for fractures with post-reduction radial short­ening >3 mm, dorsal tilt >10°, or intra-articular displacement or step-off >2 mm. A consecutive sampling method was used to minimize selection bias. Eligible patients were identified from hospital records and enrolled consecutively. A formal sample size calculation was not performed in advance due to the retrospective nature of the study. Prior to surgery, all patients who met the above surgical indications were thoroughly informed about the three operative options (BEF, EF, or VLP) and selected their treatment based on medical advice and personal preference. This study was conducted in accordance with the ethical principles of the Declaration of Helsinki and was approved by the Institutional Review Board (IRB) of the First Affiliated Hospital of Zhejiang Chinese Medical University (Approval No. 2023-KLS-356-02). Due to the retrospective design, the requirement for informed consent was waived by the IRB. Reasonable efforts have been made to anonymize individuals, and written informed consent for the publication of identifiable details has been obtained from the participants.

### Participant selection

The inclusion criteria were: (i) patients aged over 18 years with closed, unstable DRF; (ii) treatment using VLP, EF, or BEF; and (iii) minimum follow-up of 24 months.

Exclusion criteria were: (i) open or pathological fractures, fractures with neurovascular compromise or osteofascial compartment syndrome, or additional fractures in the injured limb; (ii) history of prior surgery or trauma, or pre-existing disability in the injured wrist; (iii) psychiatric illness, malignant tumors, or comorbidities affecting wrist function (e.g. osteoarthritis and infection); and (iv) incomplete data or loss to follow-up.

### Participant management

**Figure 2.**
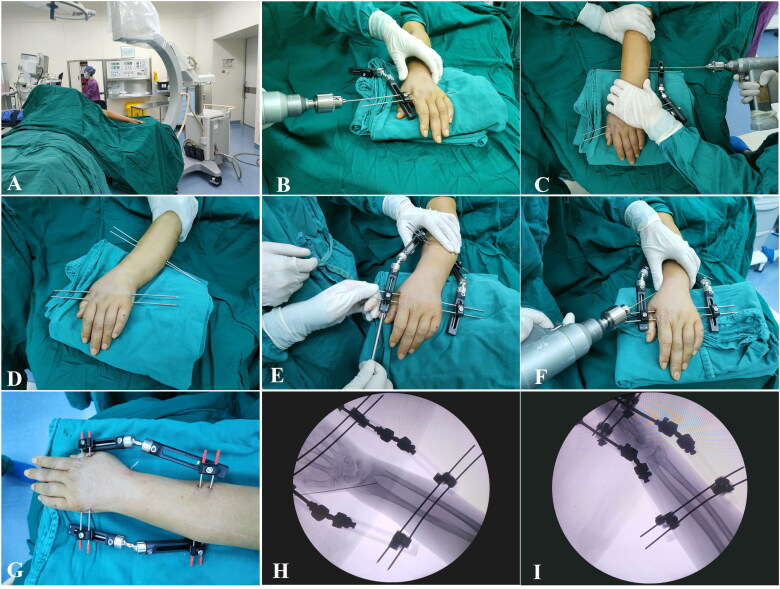
Surgical procedure for BEF application. (A) Patient positioning: supine with the injured limb placed on a radiolucent table or C-arm fluoroscopy machine. (B) Insertion of two Kirschner wires horizontally through the 2nd to 4th or 5th metacarpal bones, perpendicular to the metacarpal axis. (C) Insertion of two additional Kirschner wires horizontally through the radius. (D) Placement of all four Kirschner wires in the injured hand and forearm. (E) Application of the BEF device: two external fixators positioned on the ulnar and radial sides and secured to the Kirschner wires. Traction is applied *via* knob rotation to restore fracture alignment. (F) One or two additional 1.5 mm Kirschner wires inserted to provide supplementary fixation. (G) Postoperative clinical appearance of the BEF. (H, I) Intraoperative anteroposterior and lateral radiographs obtained using a C-arm fluoroscopy machine, confirming satisfactory restoration of volar tilt, radial inclination, radial height, and ulnar variance after fracture reduction and fixation. BEF: bi-frame external fixation; DRF: distal radius fracture.

All surgeries were performed by a single orthopedic trauma team composed of one chief surgeon (with over 15 years of clinical experience) and two senior attending surgeons (each with over 10 years of experience). Surgeries were performed under brachial plexus block anesthesia with the patient in the supine position and the injured limb placed on a radiolucent table or C-arm platform (Figure 2A).

BEF: A brief description of the surgical procedure is shown in Figure 2. Two appropriately sized Kirschner wires (diameter adapted to the external fixation chuck) were inserted horizontally through the 2nd to 4th or 5th metacarpal bones, perpendicular to the longitudinal axis of the metacarpals (Figure 2B). Similarly, two additional Kirschner wires were inserted horizontally through the radius (Figure 2C). After placement, all four Kirschner wires were confirmed to be in proper alignment (Figure 2D). The BEF device was mounted on both the radial and ulnar sides, secured to the Kirschner wires, and adjusted using the fixator knobs to restore radial length and correct fracture displacement (Figure 2E). Wrist positioning (dorsiflexion, flexion, or ulnar deviation) was modified as needed to optimize fracture alignment. Once the Kirschner wires exhibited slight elastic deformation, the device was locked in place. One or two additional 1.5 mm Kirschner wires were inserted to further enhance stability (Figure 2F). The final configuration of the BEF device is shown in Figure 2G. Intraoperative C-arm fluoroscopy was used to confirm satisfactory fracture reduction (Figure 2H, 1), and adjustments were made if necessary.

Conventional EF: Two 3 mm screws were inserted through dorsolateral incisions into the radius and second metacarpal and connected to the external fixator. After achieving satisfactory manual reduction, the fixator was then securely tightened. One or two additional 1.5 mm Kirschner wires were used to enhance fixation stability [14].

VLP: Following fracture reduction, fixation was achieved using a VLP and screws *via* the standard modified Henry approach [14].

Postoperative management included finger flexion and extension exercises initiated immediately after anesthesia recovery in the BEF and EF groups. Early wrist and finger mobilization was started in the VLP group. Following the removal of EF or BEF (approximately 6 weeks postoperatively), fist clenching, wrist extension and flexion, and progressive weight-bearing exercises were initiated. Full weight-bearing was permitted after radiographic confirmation of fracture healing.

### Data collection and outcome definition

Patients were assessed perioperatively and at follow-ups scheduled at 6 weeks, 12 weeks, 6 months, 1 year, and 2 years postoperatively. Radiographs were obtained at each follow-up, and complications were documented throughout the study.

Perioperative outcomes: Time from injury to surgery and operation time were documented.

Functional outcomes: Wrist range of motion (ROM) (flexion, extension, supination, pronation, ulnar deviation, and radial deviation) was measured using a goniometer and recorded in degrees, with higher values indicating greater mobility. Grip strength was measured using a dynamometer and recorded in kilograms (kg) [14]. Higher grip strength values indicated better recovery. For right-handed patients, grip strength on the dominant side was assumed to be 10% greater than the contralateral side; for left-handed patients, grip strength was assumed equal bilaterally [20,21].

Patient-Reported Outcomes: These included the Visual Analog Scale (VAS) for pain [11], the Patient-Rated Wrist Evaluation (PRWE) [22,23], and the Quick Disabilities of the Arm, Shoulder, and Hand (QuickDASH) [24]. Higher scores indicated worse outcomes. The VAS ranges from 0 (no pain) to 10 (worst pain). The PRWE is a 15-item scale evaluating pain and function, scored from 0 (no pain or disability) to 100 (maximum disability). QuickDASH is a shortened 11-item version of the DASH outcome measure for upper extremity disability, also scored from 0 (no disability) to 100 (most severe disability). All scores were collected for the injured side and compared between groups at each follow-up.

Radiographic outcomes: Volar tilt, radial inclination, radial height, and ulnar variance were measured preoperatively, immediately postoperatively, at 6 weeks, and at 2 years postoperatively. Ulnar styloid fracture reduction and healing were also assessed. New bone formation was evaluated through serial radiographs at each follow-up. Early-stage healing (6–12 weeks) was assessed by the presence of periosteal callus, bridging trabeculae across the fracture line, and gradual narrowing of the fracture gap. Mature bone healing was defined by complete cortical continuity and the disappearance of the fracture line. These radiographic assessments enabled longitudinal monitoring of fracture healing, with early callus formation indicating the initiation of bone regeneration and subsequent structural restoration. Additionally, they provided guidance for rehabilitation planning and adjustments during the recovery process.

Complications: Recorded complications included complex regional pain syndrome (CRPS), carpal tunnel syndrome, infection, nerve injury, radial/ulnar vascular injury, skin necrosis, fracture re-displacement, traumatic arthritis, delayed union or nonunion, scar tissue problems, tendon injury, and fixation loosening.

### Statistical analysis

Data analysis was performed using SPSS Statistics software (version 21.0; IBM Corp., Armonk, NY, USA). Continuous variables with normal distribution were expressed as mean ± standard deviation and analyzed using independent samples *t*-tests. Non-normally distributed variables were expressed as median (interquartile range, IQR) and analyzed with the Mann–Whitney *U* test. Normality was assessed using the Shapiro–Wilk test, and variance homogeneity was evaluated *via* Levene’s test. Patients were categorized into three groups: BEF, EF, and VLP. Statistical comparisons focused on pairwise analysis involving BEF (BEF vs. EF and BEF vs. VLP) to directly compare the novel technique with conventional treatments. One-way ANOVA was not used to avoid unnecessary multiple comparisons, as comparisons between EF and VLP were not the focus of this study. Categorical variables were presented as counts (%) and analyzed using Pearson’s chi-square test (with continuity correction where applicable) or Fisher’s exact test, depending on data distribution. The pairwise analytical strategy was selected to facilitate a focused comparison of BEF with each conventional treatment (EF and VLP). Statistical significance was defined as *p* < 0.05 (two-sided). The p_1_-value represented the comparison between BEF and EF, while the p_2_-value represented the comparison between BEF and VLP.

## Results

### Participant selection

**Figure 3.**
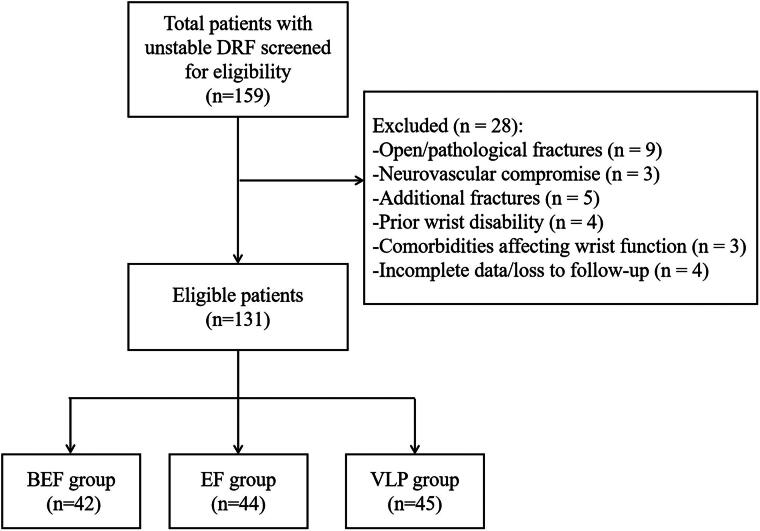
Patient enrollment flowchart.

**Figure 4.**
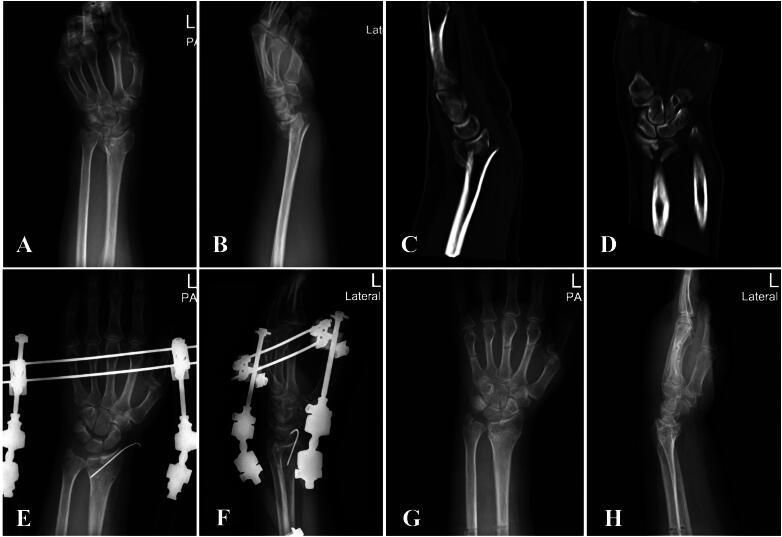
Radiographic evaluation of Case 1 treated with BEF. (A, B) Preoperative anteroposterior and lateral radiographs of a 58-year-old female patient with an AO type C2 DRF. (C, D) Preoperative coronal and sagittal CT images. (E, F) Postoperative anteroposterior and lateral radiographs showing successful fracture reduction and fixation with BEF. (G, H) Anteroposterior and lateral radiographs at 6 weeks postoperatively, showing maintained fracture alignment and early callus formation at the time of BEF removal. BEF: bi-frame external fixation; DRF: distal radius fracture.

**Figure 5.**
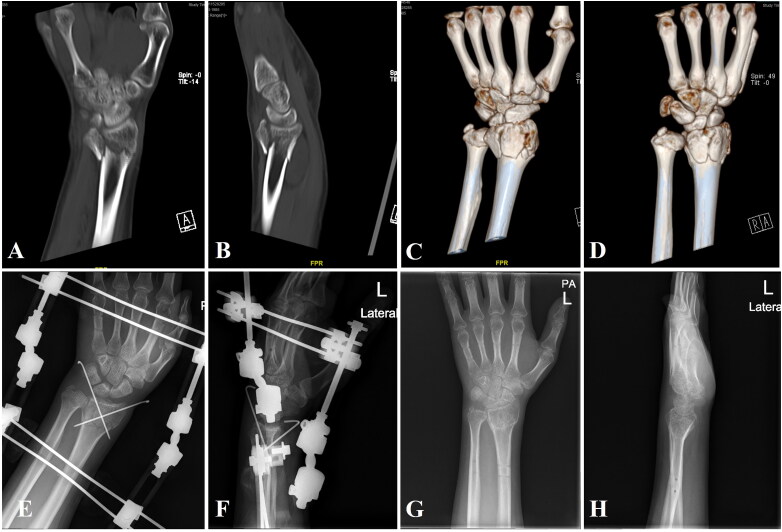
Radiographic evaluation of Case 2 treated with BEF. (A, B) Preoperative coronal and sagittal CT images of a 37-year-old male patient with an AO type C3 DRF. (C, D) Preoperative three-dimensional reconstruction of CT images illustrating complex intra-articular fracture morphology. (E, F) Postoperative anteroposterior and lateral radiographs showing successful fracture reduction and fixation with BEF. (G, H) Anteroposterior and lateral radiographs at 6 weeks postoperatively, showing maintained fracture alignment and early callus formation at the time of BEF removal. BEF: bi-frame external fixation; DRF: distal radius fracture.

A total of 159 patients were initially screened for inclusion in this study. Among them, 28 were excluded due to not meeting the eligibility criteria (e.g. open fractures, missing or incomplete follow-up data), leaving 131 patients who satisfied all inclusion and exclusion criteria. Of these, 42 patients were treated with BEF, 44 with EF, and 45 with VLP (Figure 3). Baseline characteristics – including age, sex, fracture side, and AO fracture classification – did not significantly differ between included and excluded patients (all *p* > 0.05), confirming cohort comparability. Representative BEF cases are illustrated in Figures 4 and 5. Preoperative radiographs and CT scans were used to evaluate fracture morphology (Figures 4A–D and 5A–D), while postoperative radiographs confirmed fracture reduction and fixation (Figures 4E–F and 5E–F). Follow-up radiographs obtained at 6 weeks postoperatively were used to evaluate fracture alignment and early callus formation following BEF removal (Figures 4G–H and 5G–H). Baseline characteristics and perioperative outcomes of all patients are presented in Table 1.

**Table 1.** Baseline characteristics and perioperative outcomes of patients with unstable DRF treated at the First Affiliated Hospital of Zhejiang Chinese Medical University, from February 2015 to January 2022.

Characteristic	Total (*n* = 131)	BEF (*n* = 42)	EF (*n* = 44)	VLP (*n* = 45)	*p*_1_ value	*p*_2_ value
Age (years)	46.1 ± 12.9	45.7 ± 13.3	47.1 ± 12.5	45.3 ± 13.1	0.605	0.900
Sex, *n* (%)					0.862	0.869
Male	53 (40.5%)	17 (40.5%)	17 (38.6%)	19 (42.2%)		
Female	78 (59.5%)	25 (59.5%)	27 (61.4%)	26 (57.8%)		
Fracture side, *n* (%)					0.707	0.987
Dominant side	92 (70.2%)	29 (69.0%)	32 (72.7%)	31 (68.9%)		
Non-dominant	39 (29.8%)	13 (31.0%)	12 (27.3%)	14 (31.1%)		
Fracture AO type, *n* (%)					0.917	0.852
A2	12 (9.2%)	3 (7.1%)	5 (11.4%)	4 (8.9%)		
A3	33 (25.2%)	9 (21.4%)	11 (25.0%)	13 (28.9%)		
C1	47 (35.9%)	16 (38.1%)	14 (31.8%)	17 (37.8%)		
C2	27 (20.6%)	9 (21.4%)	10 (22.7%)	8 (17.8%)		
C3	12 (9.2%)	5 (11.9%)	4 (9.1%)	3 (6.7%)		
Follow-up (months)	26.0 [25.0,27.0]	26.0 [25.0,27.0]	26.0 [25.0,27.0]	26.0 [24.0,27.0]	0.084	0.963
Time between injury and surgery (hours)	20.9 [15.1,56.6]	17.2 ± 5.2	16.9 ± 6.1	68.3 ± 24.1	0.847	**<0.001**
Operation time (minutes)	57.0 [48.0,79.0]	49.6 ± 8.4	53.9 ± 9.5	84.7 ± 14.0	**0.028**	**<0.001**

Abbreviations: DRF: distal radius fractures; BEF: bi-frame external fixation device; EF: external fixator; VLP: volar locking plate; p_1_: *p*-value for comparison between BEF and EF groups. p_2_: *p*-value for comparison between BEF and VLP groups. Values in bold denote statistical significance at *p* < 0.05.

### Participant characteristics

The study cohort (*n* = 131) had a mean age of 46.1 ± 12.9 years, with 53 males (40.5%) and 78 females (59.5%). Fractures occurred on the dominant side in 92 patients (70.2%) and on the non-dominant side in 39 (29.8%). According to the AO classification, fractures were categorized as A2 (9.2%), A3 (25.2%), C1 (35.9%), C2 (20.6%), and C3 (9.2%). The median follow-up duration was 26.0 months [IQR: 25.0–27.0]. The mean age, sex distribution, fracture side distribution (dominant or non-dominant), fracture AO type and duration of follow up were comparable between groups (Table 1).

### Perioperative outcomes

The BEF group had a significantly shorter time from injury to surgery compared to the VLP group (17.2 ± 5.2 h vs. 68.3 ± 24.1 h, *p* < 0.001) and a shorter operation time compared to both the EF group (49.6 ± 8.4 min vs. 53.9 ± 9.5 min, *p* = 0.028) and the VLP group (49.6 ± 8.4 min vs. 84.7 ± 14.0 min, *p* < 0.001) (Table 1).

### Functional outcomes

Flexion, supination, radial deviation, and grip strength: The BEF group had significantly lower flexion, supination angle, radial deviation and grip strength than the VLP group at 6 weeks and 12 weeks but these differences were not significant at other time points. The same measurements in the BEF group did not significantly differ from that in the EF group at all time points (Table 2).

**Table 2.** Functional outcomes of patients with unstable distal DRF treated at the First Affiliated Hospital of Zhejiang Chinese Medical University, from February 2015 to January 2022.

Variables	BEF(*n* = 42)	EF(*n* = 44)	VLP(*n* = 45)	*p*_1_ value	*p*_2_ value
Flexion (°)					
6 wk	29.00 [19.75,34.00]	27.00 [20.00,32.75]	48.00 [39.00,53.50]	0.653	**<0.001**
12 wk	48.93 ± 7.33	50.45 ± 8.25	57.02 ± 6.48	0.368	**<0.001**
6 mo	60.76 ± 4.51	60.55 ± 4.29	61.60 ± 5.63	0.820	0.448
1 yr	61.83 ± 4.90	60.93 ± 4.16	62.76 ± 5.05	0.360	0.390
2 yr	63.67 ± 5.07	61.86 ± 4.10	63.42 ± 5.01	0.074	0.822
Extension(°)					
6 wk	21.74 ± 7.01	19.20 ± 7.39	44.07 ± 9.19	0.107	**<0.001**
12 wk	50.95 ± 8.03	48.84 ± 9.47	54.31 ± 8.55	0.269	0.063
6 mo	56.60 ± 5.89	58.70 ± 5.85	59.91 ± 3.92	0.099	**0.003**
1 yr	60.10 ± 4.18	59.20 ± 5.97	60.78 ± 4.02	0.424	0.440
2 yr	61.24 ± 3.81	59.48 ± 5.82	63.07 ± 4.97	0.100	0.059
Supination (°)					
6 wk	26.10 ± 5.60	25.80 ± 4.95	50.24 ± 7.22	0.793	**<0.001**
12 wk	53.12 ± 9.27	50.23 ± 9.13	66.80 ± 8.69	0.149	**<0.001**
6 mo	73.00 ± 5.64	71.93 ± 5.71	74.69 ± 8.36	0.385	0.270
1 yr	77.88 ± 5.69	77.84 ± 5.03	78.09 ± 7.04	0.972	0.880
2 yr	82.00 ± 5.02	81.75 ± 5.35	82.93 ± 3.99	0.824	0.338
Pronation (°)					
6 wk	28.17 ± 5.53	27.00 ± 4.26	52.56 ± 9.07	0.275	**<0.001**
12 wk	52.36 ± 7.05	44.57 ± 8.77	62.00 ± 11.07	**<0.001**	**<0.001**
6 mo	75.40 ± 7.31	71.34 ± 6.41	77.00 ± 7.34	**0.007**	0.313
1 yr	78.52 ± 5.89	75.64 ± 4.09	79.00 ± 6.93	**0.010**	0.732
2 yr	79.40 ± 5.02	77.07 ± 4.41	80.80 ± 6.37	**0.024**	0.262
Ulnar deviation (°)					
6 wk	18.21 ± 5.45	17.07 ± 5.76	24.64 ± 5.71	0.347	**<0.001**
12 wk	23.45 ± 5.40	20.84 ± 7.23	31.36 ± 8.39	0.062	**<0.001**
6 mo	37.00 [31.75,40.00]	34.00 [31.00,39.00]	35.00 [31.50,40.50]	0.208	0.925
1 yr	39.90 ± 4.88	37.11 ± 5.57	39.49 ± 6.63	**0.016**	0.741
2 yr	41.02 ± 5.28	37.82 ± 6.10	40.02 ± 6.87	**0.011**	0.450
Radial deviation (°)					
6 wk	−1.40 ± 7.87	−3.70 ± 6.80	12.69 ± 6.65	0.150	**<0.001**
12 wk	14.79 ± 5.53	13.95 ± 4.38	19.42 ± 4.95	0.441	**<0.001**
6 mo	23.67 ± 5.36	22.57 ± 5.79	23.76 ± 4.23	0.364	0.932
1 yr	25.40 ± 5.77	24.66 ± 4.89	26.02 ± 4.06	0.519	0.568
2 yr	27.00 [20.00,31.00]	24.50 [22.00,28.00]	27.00 [25.00,29.00]	0.380	0.795
Grip strength (kg)					
6 wk	2.00[1.00,3.00]	2.00[2.00,3.00]	13.00 [8.50,17.00]	0.267	**<0.001**
12 wk	12.10 ± 4.25	12.95 ± 4.20	20.36 ± 7.10	0.348	**<0.001**
6 mo	28.69 ± 6.07	29.25 ± 5.92	29.44 ± 5.84	0.666	0.557
1 yr	39.00 [30.75,43.00]	37.00 [32.25,41.75]	39.00 [33.00,42.50]	0.508	0.858
2 yr	39.00 ± 6.42	37.11 ± 6.23	40.18 ± 5.15	0.170	0.347

Abbreviations: DRF: distal radius fractures; BEF: bi-frame external fixation device; EF: external fixator; VLP: volar locking plate; p_1_: p-value for comparison between BEF and EF groups. p_2_: p-value for comparison between BEF and VLP groups. Values in bold denote statistical significance at *p* < 0.05.

Extension: The BEF group had significantly lower extension compared to the VLP group at 6 weeks (21.74 ± 7.01° vs. 44.07 ± 9.19°, *p* < 0.001) and 6 months (56.60 ± 5.89° vs. 59.91 ± 3.92°, *p* = 0.003), but these differences were not significant at other time points. Extension in the BEF group was not significantly different from that in the EF group throughout the follow-up (Table 2).

Pronation: The BEF group had significantly lower pronation compared to the VLP group at 6 weeks (28.17 ± 5.53° vs. 52.56 ± 9.07°, *p* < 0.001) and 12 weeks (52.36 ± 7 .05° vs. 62.00 ± 11.07°, *p* < 0.001), but these differences were not significant at other time points. The BEF group had significantly better pronation compared to the EF group at 12 weeks (52.36 ± 7.05° vs. 44.57 ± 8.77°, *p* < 0.001), 6 months (75.40 ± 7.31° vs. 71.34 ± 6.41°, *p* = 0.007), 1 year (78.52 ± 5.89° vs. 75.64 ± 4.09°, *p* = 0.010), and 2 years (79.40 ± 5.02° vs. 77.07 ± 4.41°, *p* = 0.024).

Ulnar deviation: The BEF group had significantly lower ulnar deviation compared to the VLP group at 6 weeks (18.21 ± 5.45° vs. 24.64 ± 5.71°, *p* < 0.001) and 12 weeks (23.45 ± 5 .40° vs. 31.36 ± 8.39°, *p* < 0.001), but these differences were not significant at other time points. The BEF group had significantly higher ulnar deviation compared to the EF group at 1 year (39.90 ± 4.88° vs. 37.11 ± 5.57°, *p* = 0.016) and 2 years (41.02 ± 5.28° vs. 37.82 ± 6.10°, *p* = 0.011) (Table 2).

**Figure 6.**
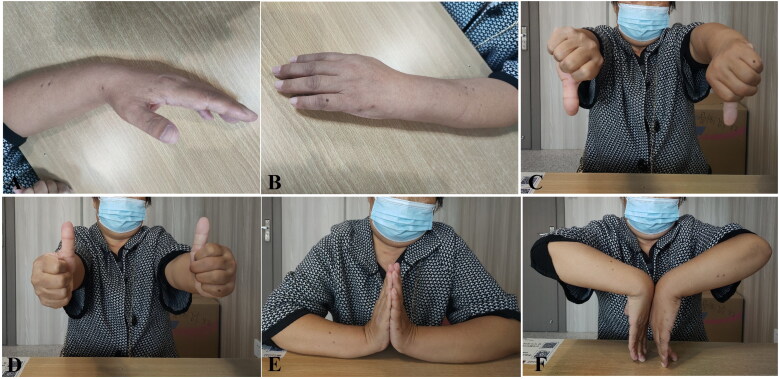
Operative area appearance and postoperative wrist ROM of Case 1 (Figure 3) at 12-week follow-up.(A, B) Clinical appearance of the operative area. (C–F) Functional assessment of wrist motion, including pronation (C), neutral position (D), extension (E), and flexion (F), demonstrating satisfactory functional recovery at 12 weeks postoperatively.ROM: range of motion.

**Figure 7.**
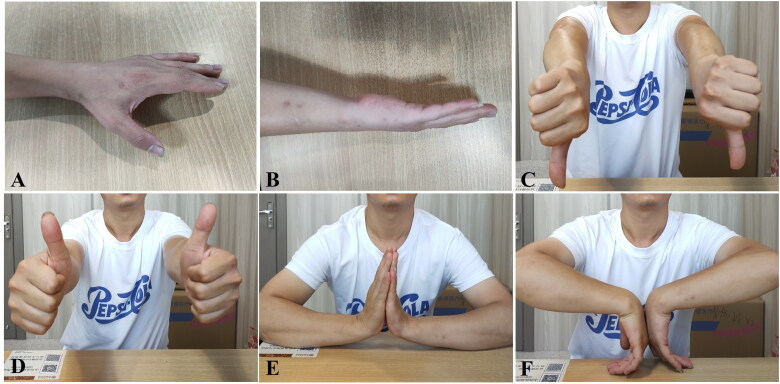
Operative area appearance and postoperative wrist ROM of Case 2 (Figure 4) at 12-week follow-up. (A, B) Clinical appearance of the operative area. (C–F) Functional assessment of wrist motion, including pronation (C), neutral position (D), extension (E), and flexion (F), demonstrating satisfactory functional recovery at 12 weeks postoperatively. ROM: range of motion.

Figures 6 and 7 illustrate the operative area appearance (Figures 6A,B and 7A,B) and postoperative wrist ROM (Figures 6C–F and 7C–F) in representative BEF cases at 12 weeks.

### Patient-reported outcomes

VAS scores did not significantly differ between the BEF group and either the VLP or EF groups at any time point. Compared to the VLP group, the BEF group exhibited significantly higher PRWE scores at 6 weeks (46.00 ± 6.56 vs. 30.51 ± 5.02, *p* < 0.001) and 12 weeks (27.00 [24.75–30.00] vs. 19.00 [16.50–21.50], *p* < 0.001), and higher QuickDASH scores at 6 weeks (47.05 ± 10.37 vs. 37.02 ± 8.50, *p* < 0.001) and 12 weeks (29.07 ± 8.23 vs. 20.98 ± 7.29, *p* < 0.001). No significant differences in these scores were observed at later follow-ups. The same scores did not significantly differ between the BEF and EF groups at any time point (Table 3).

**Table 3.** Patient-reported outcomes of patients with unstable DRF treated at the First Affiliated Hospital of Zhejiang Chinese Medical University, from February 2015 to January 2022.

Variables	BEF (*n* = 42)	EF (*n* = 44)	VLP (*n* = 45)	*p*_1_ value	*p*_2_ value
Pain (VAS)					
6 wk	2.00 [1.00,2.00]	2.00 [1.00,2.00]	2.00 [1.00,3.00]	0.405	0.300
12 wk	1.00 [1.00,2.00]	1.00 [1.00,2.00]	2.00 [1.00,3.00]	0.775	0.179
6 mo	1.00 [1.00,1.00]	1.00 [1.00,1.00]	1.00 [1.00,1.00]	0.841	0.860
1 yr	1.00 [0.00,1.00]	1.00 [0.00,1.00]	1.00 [0.00,1.00]	0.713	0.973
2 yr	1.00 [0.00,1.00]	1.00 [0.00,1.00]	1.00 [0.00,1.00]	0.841	0.930
PRWE score					
6 wk	46.00 ± 6.56	47.02 ± 6.65	30.51 ± 5.02	0.475	**<0.001**
12 wk	27.00 [24.75,30.00]	29.00[25.50,31.00]	19.00[16.50,21.50]	0.097	**<0.001**
6 mo	13.45 ± 4.04	14.05 ± 4.51	13.89 ± 4.00	0.523	0.614
1 yr	6.00 [4.00,8.00]	6.00 [5.00,7.00]	6.00 [4.50,8.00]	0.560	0.925
2 yr	5.67 ± 2.28	5.70 ± 1.69	5.51 ± 1.72	0.930	0.720
QuickDASH score					
6 wk	47.05 ± 10.37	48.05 ± 10.69	37.02 ± 8.50	0.662	**<0.001**
12wk	29.07 ± 8.23	30.00 ± 8.91	20.98 ± 7.29	0.617	**<0.001**
6mo	17.71 ± 6.12	18.00 ± 5.78	17.82 ± 5.12	0.824	0.929
1yr	8.19 ± 3.54	8.48 ± 3.79	8.33 ± 3.34	0.718	0.847
2yr	7.40 ± 3.44	7.61 ± 3.34	7.02 ± 3.12	0.776	0.587

Abbreviations: DRF: distal radius fractures; BEF: bi-frame external fixation device; EF: external fixator; VLP: volar locking plate; VAS: visual analog scale; PRWE: patient-rated wrist evaluation; QuickDASH: quick disabilities of the arm, shoulder, and hand; p_1_: *p*-value for comparison between BEF and EF groups. p_2_: *p*-value for comparison between BEF and VLP groups. Values in bold denote statistical significance at *p* < 0.05.

### Radiographic outcomes

Preoperative radiographic parameters (volar tilt, radial inclination, radial height, ulnar variance) were comparable between groups (all *p* > 0.05). Postoperatively, the BEF group exhibited superior radial height and ulnar variance compared to the EF group at all follow-up time points (immediately postoperatively, 6 weeks, and 2 years), whereas no differences were observed in volar tilt or radial inclination between the two groups. Comparisons between the BEF and VLP groups revealed no significant differences in any radiographic parameters at any time point (Table 4).

**Table 4.** Radiographic outcomes of patients with unstable DRF treated at the First Affiliated Hospital of Zhejiang Chinese Medical University, from February 2015 to January 2022.

Variables	BEF(*n* = 42)	EF(*n* = 44)	VLP(*n* = 45)	*p*_1_ value	*p*_2_ value
Volar tilt (°)					
Preoperative	−21.50 ± 9.57	−20.89 ± 8.59	−22.00 ± 10.25	0.755	0.815
Postoperative	5.00 ± 4.50	4.50 ± 3.97	5.80 ± 4.14	0.586	0.390
6 wk	4.90 ± 4.10	3.84 ± 3.95	5.60 ± 4.02	0.224	0.427
2 yr	5.21 ± 4.08	4.25 ± 4.03	5.51 ± 3.88	0.273	0.729
Radial inclination(°)					
Preoperative	10.60 ± 4.57	10.50 ± 3.26	11.02 ± 3.04	0.911	0.612
Postoperative	20.81 ± 3.23	19.61 ± 3.79	21.51 ± 2.71	0.120	0.275
6 wk	20.62 ± 3.44	19.34 ± 3.70	21.20 ± 2.59	0.101	0.374
2 yr	21.00 ± 3.41	19.73 ± 3.74	21.31 ± 2.65	0.103	0.635
Radial height (°)					
Preoperative	5.81 ± 2.35	6.00 ± 3.52	6.20 ± 3.24	0.768	0.520
Postoperative	11.00 [11.00,12.00]	10.00 [9.00,11.00]	11.00 [9.50,13.00]	**<0.001**	0.735
6 wk	11.00 [10.00,12.00]	9.00 [9.00,10.00]	11.00 [9.00,12.00]	**<0.001**	0.521
2 yr	11.00 [10.00,12.00]	9.50 [9.00,10.00]	11.00 [9.00,12.00]	**0.004**	0.274
Ulnar variance (mm)					
Preoperative	2.81 ± 0.55	2.90 ± 0.91	3.00 ± 0.78	0.568	0.185
Postoperative	−0.60 ± 0.45	−0.25 ± 0.61	−0.56 ± 0.52	**0.003**	0.637
6 wk	−0.40[-1.00,-0.08]	0.10[-0.10,0.28]	−0.50[-0.80,-0.15]	**<0.001**	0.762
2 yr	−0.55 ± 0.49	0.20 ± 0.45	−0.54 ± 0.45	**<0.001**	0.921
Ulnar styloid fracture before operation, *n*(%)	16 (38.10%)	18 (40.91%)	20 (44.44%)	0.790	0.548
Anatomic reduction of ulnar styloid fracture, *n*(%)	6 (37.50%)	4 (22.22%)	4 (20.00%)	0.457	0.285
Ulnar styloid fracture healing at 2 yr, *n*(%)	8 (50.00%)	6 (33.33%)	5 (25.00%)	0.487	0.169

Abbreviations: DRF: distal radius fractures; BEF: bi-frame external fixation device; EF: external fixator; VLP: volar locking plate; p_1_: p-value for comparison between BEF and EF groups. p_2_: p-value for comparison between BEF and VLP groups. Values in bold denote statistical significance at* p* < 0.05.

The incidence of preoperative ulnar styloid fractures was comparable between groups (BEF: 38.1%, EF: 40.9%, VLP: 44.4%; *p* > 0.05 for BEF vs. EF and BEF vs. VLP). Among patients with ulnar styloid fractures, the BEF group had a higher anatomical reduction rate (37.5%) than the EF (22.2%) and VLP (20.0%) groups, though these differences were not statistically significant. At 2 years, the BEF group exhibited a higher healing rate for ulnar styloid fractures (50.0%) than the EF (33.3%) and VLP (25.0%) groups, but again, the differences were not statistically significant (Table 4).

### Complications

The BEF group had significantly fewer overall complications compared to the EF group (4.76% vs. 27.27%, *p* = 0.005), while no significant differences were observed between BEF and VLP groups (4.76% vs. 15.56%, *p* = 0.194) (Table 5).

**Table 5.** Complications at 2-year follow-up in patients with unstable DRF treated at the First Affiliated Hospital of Zhejiang Chinese Medical University, from February 2015 to January 2022.

Complications	No. of patients				
	BEF (*n* = 42)	EF (*n* = 44)	VLP (*n* = 45)	*p*_1_ value	*p*_2_ value
Overall complications, *n* (%)	2 (4.76%)	12 (27.27%)	7 (15.56%)	**0.005**	0.194
Specific compli­cations, *n* (%)					
Complex regional pain syndrome	1 (2.38%)	3 (6.82%)	0	0.642	0.483
Carpal tunnel syndrome	0	0	0	–	–
Infection	0	1 (2.27%)	1 (2.22%)	1.000	1.000
Nerve injury	0	0	1 (2.22%)	–	1.000
Radial/ulnar vascular injury	0	0	2 (4.44%)	–	0.505
Skin necrosis	0	0	0	–	–
Fracture re-displacement	0	2 (4.55%)	0	0.495	–
Traumatic arthritis	1 (2.38%)	3 (6.82%)	1 (2.22%)	0.642	1.000
Delayed union or nonunion	0	0	0	–	–
Scar tissue problems	0	0	1 (2.22%)	–	1.000
Tendon injury	0	0	1 (2.22%)	–	1.000
Fixation loosening	0	3 (6.82%)	0	0.257	–

Abbreviations: DRF: distal radius fractures; BEF: bi-frame external fixation device; EF: external fixator; VLP: volar locking plate; p_1_: p-value for comparison between BEF and EF groups. *p*_2_: *p*-value for comparison between BEF and VLP groups. All *p*-values were derived from Pearson’s chi-square tests (with continuity correction when necessary) or Fisher’s exact tests, as appropriate for the distribution of categorical variables. Values in bold denote statistical significance at *p* < 0.05.

In the BEF group, CRPS and traumatic arthritis each occurred in 1 case (2.38%). In the EF group, superficial infection occurred in 1 case (2.27%), fracture re-displacement in 2 cases (4.55%), and CRPS, traumatic arthritis, and fixation loosening each occurred in 3 cases (6.82%). In the VLP group, infection, nerve injury, traumatic arthritis, tendon injury, and scar tissue problems each occurred in 1 case (2.22%), while radial/ulnar vascular injury occurred in 2 cases (4.44%). No significant differences were observed in the incidence of specific complications between groups (Table 5).

## Discussion

This study aimed to evaluate the clinical efficacy and safety of a novel BEF for unstable DRF by comparing it with the VLP and EF. The main findings are as follows: (1) The BEF group had a significantly shorter time from injury to surgery than the VLP group, and a shorter operative duration than both EF and VLP. (2) Short-term wrist ROM and grip strength were lower in the BEF group than in the VLP group, but no significant differences were observed in the long term. Additionally, BEF demonstrated significantly greater pronation and ulnar deviation than EF at long-term follow-up. (3) Short-term PRWE and QuickDASH scores were higher (worse) in the BEF group than in the VLP group, but these differences disappeared in the long term. These scores did not differ significantly between BEF and EF at any time point, and VAS scores were similar between groups. (4) BEF provided superior restoration and maintenance of radial height and ulnar variance compared to EF, while radiographic outcomes were comparable between BEF and VLP. (5) The overall complication rate was significantly lower in the BEF group than in the EF group and comparable to that of the VLP group. Specific complications did not differ significantly between groups.

The cohort characteristics in this study align with the typical demographics of patients with unstable DRF, including a mean age of 46.1 years and a near-equal sex distribution (40.5% male, 59.5% female). This is consistent with the known epidemiology of DRF, which commonly affects both younger individuals (due to high-energy trauma) and older adults (due to osteoporotic low-energy trauma) [3–5]. The inclusion of a broad range of AO fracture types (A2–C3) (A2-C3), covering both extra-articular and complex intra-articular fractures, further enhances the generalizability of the findings. Consecutive patient enrollment over a seven-year period also reduced selection bias. Moreover, no significant differences in baseline characteristics were observed between excluded and included patients, strengthening the representativeness of the final cohort.

Baseline variables, including age, sex, fracture side, and AO classification, were well balanced between groups, minimizing selection bias and ensuring comparability. While these variables were evenly distributed, patient-specific factors such as fracture complexity and bone quality may still influence outcomes. To minimize confounding effects, strict inclusion and exclusion criteria were applied, and all surgeries were performed by a single orthopedic trauma team using a standardized surgical and rehabilitation protocol.

In this study, the BEF group demonstrated a significantly shorter time from injury to surgery compared to the VLP group, as well as a shorter operative duration than both the EF and VLP groups. These findings are consistent with those reported by Zhang et al. [14], who observed a time from injury to surgery of 14.6 ± 6.8 h for EF and 65.9 ± 23.2 h for VLP, with operative times of 62 ± 10 min and 79 ± 13 min, respectively. In contrast, Hammer et al. [25] reported longer intervals – 7.0 ± 4.7 days for EF and 6.5 ± 4.0 days for VLP – likely due to variations in preoperative management protocols across institutions. Unlike VLP, BEF does not require soft tissue detumescence prior to surgery, which contributes to its shorter time from injury to surgery. Additionally, BEF’s minimally invasive design and technical simplicity result in reduced operative duration compared to VLP. The minimally invasive nature of BEF eliminates the need for a tourniquet, thereby reducing the risk of ischemia-reperfusion injury and nerve damage associated with VLP. The bidirectional traction mechanism of BEF provides effective, sustained, and symmetrical radioulnar traction forces, facilitating faster fracture reduction and further reducing operative time compared to EF. This innovation streamlines the reduction process and eliminates some time-consuming steps required in VLP, resulting in a more efficient surgical workflow.

Although the BEF group exhibited lower wrist ROM and grip strength in the early postoperative period compared to VLP, these differences were no longer significant at longer-term follow-ups. This trend is consistent with findings from previous studies comparing EF and VLP for the treatment of DRF. Ludvigsen et al. [10] reported superior early ROM and grip strength with VLP compared to EF at 6 weeks and 3 months, but no differences at one year. Similarly, Hammer et al. [25] showed that while VLP provided early functional advantages, these had diminished by the two-year follow-up. Zhang et al. [14] reported superior wrist extension and pronation with VLP at 6 months, while Saving et al. [26] found no long-term differences in grip strength or ROM between VLP and EF at three years. These results collectively support the view that VLP enables faster early functional recovery, whereas long-term outcomes tend to converge across fixation methods. The early ROM and grip strength disadvantages of BEF may be attributed to the temporary wrist immobilization required postoperatively, in contrast to the immediate mobilization permitted with VLP. Nonetheless, our study demonstrated that after BEF removal, wrist ROM and grip strength improved progressively, eventually reaching levels comparable to those of the VLP group. Furthermore, BEF showed significantly better pronation and ulnar deviation than EF at long-term follow-up, likely due to its biomechanical advantage in maintaining symmetrical and sustained traction.

Patient-reported outcome measures in this study followed a similar trend to the functional outcomes. The BEF group showed higher (worse) PRWE and QuickDASH scores than the VLP group at early follow-up stages; however, these differences were no longer statistically significant at long-term follow-up. This finding is consistent with previous studies comparing VLP and EF in the treatment of DRF, which also reported better early patient-reported outcomes with VLP but no significant differences in the long term [6,10, 14,25–27]. These findings suggest that, despite initial delays in recovery, BEF ultimately provides comparable levels of independence in daily activities and quality of life as VLP. Moreover, PRWE, QuickDASH, and VAS scores did not significantly differ between BEF and EF at any time point.

Radiographic assessments revealed no significant differences in outcomes between the BEF and VLP groups across all measured time points. In contrast, EF was less effective in restoring and maintaining radial length and ulnar variance compared to BEF, underscoring the efficacy of BEF in DRF management and its superiority over EF in fracture reduction. These findings are consistent with previous studies highlighting the limitations of EF in maintaining these radiographic parameters. Hammer et al. [25], Mellstrand et al. [28], Williksen et al. [29], and Karantana et al. [30] reported that EF was inferior to VLP in restoring and maintaining radial height, while Ludvigsen et al. [10] and Williksen et al. [29,31] similarly found that EF was less effective than VLP in restoring and preserving ulnar variance. Our results further support the notion that BEF can mitigate these shortcomings of EF, achieving radiographic outcomes comparable to those of VLP. Notably, the BEF group exhibited a higher rate of anatomical reduction and healing of ulnar styloid fractures than both the EF and VLP groups, whereas the VLP group had the lower healing rate than the BEF group. The reduced healing rate in the VLP group may be related to the earlier initiation of functional exercises, which could interfere with fracture healing. However, these differences related to ulnar styloid fractures were not statistically significant, indicating that although BEF may provide improved stability and fracture management, further studies are needed to validate its potential advantages in achieving anatomical reduction and promoting healing of ulnar styloid fractures associated with DRF.

The BEF group experienced a significantly lower overall complication rate compared to the EF group (4.76% vs. 27.27%, *p* = 0.005), while no significant difference was observed between the BEF and VLP groups (4.76% vs. 15.56%, *p* = 0.194). Given the minimally invasive nature of BEF relative to VLP, our findings suggest a trend toward fewer surgery-related complications, including nerve injury, vascular injury, scar tissue formation, and tendon damage in the BEF group. In addition, the superior stability and effective reduction achieved by BEF, compared to EF, may explain the lower incidence of fracture re-displacement, traumatic arthritis, and fixation loosening in the BEF group.While the limited sample size may have reduced the statistical power to detect differences in specific complications, the significantly lower overall complication rate still highlights the potential clinical benefits of BEF. Prior studies have reported higher complication rates with EF compared to VLP, primarily due to inadequate fixation stability, which often results in reduction loss and the need for secondary procedures. In contrast, VLP is more often associated with open surgical complications such as median nerve palsy, tenosynovitis, and tendon rupture [25,32,33]. Our findings are consistent with this established complication profile.

The biomechanical superiority of BEF over both VLP and EF lies in its ability to balance mechanical stability with mechanobiological stimulation. It avoids the excessive rigidity associated with VLP, which can inhibit callus formation, as well as the insufficient stability of EF, which increases the risk of fracture displacement. The bi-frame design of BEF generates symmetrical traction forces that are counterbalanced by the elastic deformation of the Kirschner wires, forming a closed-loop system. This configuration minimizes shear stress while allowing controlled micromotion, thereby promoting secondary bone healing. Unlike VLP, which immobilizes fractures through direct plate-to-bone contact and often induces stress shielding, BEF relies on ligamentotaxis and soft tissue compression to maintain interfragmentary strain and create a biologically favorable environment for callus formation [17,18]. Compared to traditional EF, which delivers unidirectional traction and may lead to asymmetric loading, BEF’s bi-frame configuration provides multidirectional stability. This helps reduce focal stress concentrations at the pin–bone interface and mitigates the risk of pin loosening and loss of reduction, especially in osteoporotic bone. Furthermore, the ‘bowstring effect’ produced by the tensioned Kirschner wires enhances primary mechanical stability while preserving periosteal blood supply, which is essential for osteocyte survival and effective fracture healing.

The adjustability of the BEF system allows surgeons to optimize its configuration according to the specific fracture geometry, which is a critical determinant of mechanical stability and healing outcomes. Fracture gap size directly affects the mechanical microenvironment at the healing site; smaller gaps promote osteoblast differentiation, while larger gaps can result in excessive interfragmentary strain, potentially leading to fibrous tissue formation [34]. The BEF’s bi-frame design enables precise modulation of traction forces through controlled rotation of the radial and ulnar knobs, combined with strategic wrist positioning (e.g. dorsiflexion, flexion, or ulnar deviation). This adaptability allows for individualized adjustment based on fracture characteristics such as gap size, displacement direction, and fragment angulation. For example, increasing traction *via* the knobs can restore radial length and correct fragment displacement, while wrist positioning adjustments can help optimize volar tilt and radial inclination. By dynamically balancing interfragmentary strain and maintaining physiological alignment, BEF fosters a biomechanical environment that supports fracture healing, consistent with mechanobiological principles linking controlled mechanical stimuli to favorable cellular responses [34]. This geometric adaptability not only improves reduction accuracy but also reduces the risk of re-displacement, particularly in complex or unstable fracture patterns, highlighting the versatility of BEF in managing a wide range of fracture geometries.

The primary risk associated with our innovative technique is the potential for Kirschner wire insertion to cause injury to peripheral nerves and blood vessels. To minimize this risk, we manually aligned the 2nd to 5th metacarpals at an even level before carefully inserting the Kirschner wires horizontally through the 2nd to 4th or 5th metacarpal bones, ensuring that the wires were positioned away from neurovascular structures. Kirschner wires were primarily inserted through the 2nd and 3rd metacarpals, as their force vectors align with the radial direction. Additionally, to further protect the ulnar nerve, two additional Kirschner wires were placed across the radius from the ulnar to the radial side. The accuracy of wire placement was confirmed intraoperatively using C-arm fluoroscopy. This method is similar to the established metacarpal traction technique. Another consideration with our innovative technique is the relatively inconvenient postoperative care required during the fixation period, which may affect patient comfort and compliance.

### Quality of recommendations and strength of evidence

The current clinical practice guideline jointly published by the American Academy of Orthopaedic Surgeons (AAOS) and the American Society for Surgery of the Hand (ASSH) provides strong evidence that long-term radiographic and patient-reported outcomes are comparable among fixation techniques for unstable DRF, including VLP, EF, and percutaneous pinning [35]. Notably, based on six high-quality studies, the guideline concludes that VLP facilitates earlier functional recovery within the first three months; however, this short-term benefit tends to diminish over time [35]. In addition, the guideline presents moderate-level evidence supporting surgical fixation in nongeriatric adults who exhibit significant post-reduction deformities, such as radial shortening >3 mm, dorsal tilt >10°, or intra-articular step-off >2 mm [35]. These indications specifically apply to nongeriatric adults, which aligns well with the demographic characteristics of our study population, as the cohort had a mean age of 46.1 ± 12.9 years.

These recommendations underscore the importance of individualized treatment planning based on patient-specific factors such as age, fracture morphology, and functional expectations. Within this context, our findings suggest that BEF achieves long-term functional and radiographic outcomes comparable to those of VLP, while surpassing EF in terms of alignment preservation, range of motion, and complication rates. Moreover, BEF enables earlier surgical intervention and shorter operative duration, making it a potentially advantageous option in clinical situations where delayed plating or soft tissue conditions may preclude the use of VLP. Collectively, our findings are consistent with and may extend existing guideline recommendations by supporting the use of BEF as a viable treatment alternative for unstable DRF.

To further assess the quality of our evidence, we applied the GRADE (Grading of Recommendations Assessment, Development and Evaluation) framework [36,37]. Due to the retrospective nature of the study, lack of randomization, and the potential for selection bias, the initial certainty of evidence was rated as low. Nevertheless, the consistency of outcome trends across groups, the presence of clinically meaningful effect sizes, and the well-balanced baseline characteristics observed in our cohort support an upgrade to moderate certainty for selected outcome comparisons. The GRADE system offers a transparent and structured method for evaluating non-randomized evidence, aiding in the interpretation of clinical relevance and applicability.

While our findings are promising, limitations such as the single-center design and modest sample size must be acknowledged. Future prospective, multicenter randomized trials are needed to confirm the efficacy and broader applicability of BEF in DRF management.

### Strengths and limitations

This study possesses several strengths. First, the balanced distribution of baseline characteristics (age, sex, AO fracture type and fracture side) between groups minimized selection bias and ensured comparability in outcome analyses. Second, the inclusion of a wide range of fracture patterns (AO types A2-C3) ensured that the study population was representative of real-world clinical cases, while consecutive enrollment over a seven-year period minimized selection bias and enhanced external validity. These factors improve the representativeness of the findings within similar clinical settings, though broader validation across multiple institutions remains necessary. Third, all procedures were performed by a single experienced orthopedic team using standardized surgical techniques and rehabilitation protocols, which minimized potential confounding factors and reduced variability in surgical execution and postoperative care. These methodological strengths and the consistency of the patient cohort contribute to the overall reliability and internal validity of the study findings.

Nevertheless, this study has several limitations. First, its retrospective nature may introduce inherent selection and information biases and limit the ability to establish causal relationships. Although we attempted to mitigate bias by enrolling all eligible patients consecutively over a seven-year period and by using standardized protocols within a single surgical team, there remains the possibility that some effects were underestimated or overestimated. Prospective randomized controlled trials remain necessary for stronger causal inferences. Second, the relatively small sample size, although sufficient to detect some statistically significant differences, may have reduced the statistical power to detect differences in less frequent outcomes (e.g. anatomical reduction and healing rates of ulnar styloid fractures, specific complications), potentially leading to underestimation or overestimation of the true effect. Additionally, as this study was conducted in a single-center setting, variations in perioperative practices across institutions may affect the reproducibility of the findings, thereby limiting external generalizability. Although internal validation was ensured, external validation across multiple centers was not performed. Future multicenter studies with larger and more diverse cohorts are warranted to improve the robustness and broader applicability of the conclusions. Third, the two-year follow-up period may not be sufficient to detect late-onset complications such as post-traumatic arthritis, potentially leading to an underestimation of long-term risks. Extending the follow-up duration in future studies would allow for a more comprehensive assessment of long-term outcomes. Therefore, prospective multicenter randomized controlled trials with longer follow-up periods are warranted to validate and expand upon these findings.

## Conclusion

This study demonstrates that the BEF system significantly reduced the time from injury to surgery compared to VLP, and shortened operative duration relative to both VLP and EF, suggesting a more streamlined surgical workflow. Although BEF was initially associated with lower wrist range of motion, reduced grip strength, and higher (worse) PRWE and QuickDASH scores than VLP in the early postoperative period, these differences were no longer significant at long-term follow-up, and radiographic outcomes remained comparable across all time points. Compared with EF, BEF provided superior restoration and maintenance of radial height and ulnar variance, along with improved long-term pronation and ulnar deviation, while PRWE and QuickDASH scores remained similar throughout the study period. Furthermore, BEF was associated with a significantly lower overall complication rate than EF and a similar rate to that of VLP, with no notable differences in specific complications or VAS scores between groups. These findings are consistent with previous reports indicating that although VLP may facilitate faster early functional recovery, long-term outcomes between VLP and EF tend to converge. By incorporating a bidirectional traction mechanism that provides stable fixation while maintaining minimal invasiveness, the BEF system appears to address key limitations of both traditional EF and open reduction with VLP, while preserving clinical efficacy and safety. This study highlights the clinical feasibility and potential benefits of BEF as a promising treatment modality for unstable DRF in adults. Nevertheless, further validation through large-scale, multicenter, prospective randomized controlled trials with extended follow-up is required to confirm these findings and evaluate the broader applicability of BEF in diverse clinical settings.

## Data Availability

The data are available from the corresponding author on reasonable request.
